# Relapsed Waldenström macroglobulinemia presenting as bilateral parotid gland enlargement with multiple cystic lesions: A case report

**DOI:** 10.1097/MD.0000000000051176

**Published:** 2026-10-23

**Authors:** Hsin-Yao Hung, Yu-Cheng Chang, Tsung-Yu Yen

**Affiliations:** a Department of Radiation Oncology, MacKay Memorial Hospital, Taipei City, Taiwan; b Department of Internal Medicine, Division of Hematology and Oncology, MacKay Memorial Hospital, Taipei City, Taiwan.

**Keywords:** extramedullary disease, parotid gland enlargement, Waldenström macroglobulinemia

## Abstract

**Rationale:**

Waldenström macroglobulinemia (WM) is a rare lymphoplasmacytic malignancy, and extramedullary disease involving the parotid glands is an exceptionally uncommon presentation.

**Patient concerns:**

We report the case of a 79-year-old man with a history of WM who had achieved stable disease for several years following multiple courses of chemotherapy. He subsequently presented with bilateral parotid gland enlargement suspicious for relapsed WM.

**Diagnoses:**

Imaging studies revealed multiple cystic lesions, and relapsed WM was considered the most likely diagnosis based on markedly elevated serum immunoglobulin M (IgM) levels and polymerase chain reaction analysis of fine-needle aspirates demonstrating immunoglobulin heavy chain (IgH) gene rearrangement.

**Interventions:**

Given his poor clinical condition, the patient received palliative care.

**Outcomes:**

The patient died due to sepsis 4 months after the presentation of parotid enlargement.

**Lessons:**

This case highlights the importance of considering relapsed WM in the differential diagnosis of parotid gland enlargement, particularly in patients with a prior history of lymphoplasmacytic malignancy.

## 1. Introduction

Waldenström macroglobulinemia (WM) is a rare lymphoplasmacytic malignancy characterized by the proliferation of lymphoplasmacytic cells in the bone marrow and the overproduction of immunoglobulin M (IgM). Its clinical manifestations are diverse and commonly include anemia, bleeding tendency, hepatosplenomegaly, and hyperviscosity syndrome.^[1]^ The diagnosis and treatment of WM are challenging, and some patients may present with atypical features. The uniqueness of this case lies in the relapse pattern, manifesting as bilateral, painful parotid gland enlargement. Imaging studies revealed multiple cystic lesions, an uncommon imaging appearance that was highly suggestive of possible extramedullary disease (EMD), underscoring the heterogeneity of organ involvement in this disease and the complexity of its diagnosis.

## 2. Case presentation

The patient first presented to our hospital in 2017, at the age of 74 years, with a one-day history of melena. During the workup for gastrointestinal bleeding, later confirmed to be caused by a gastric ulcer, we noted lymphocytosis with 14% atypical lymphocytes. A hematologist was consulted for further management; a series of tests including bone marrow survey, protein electrophoresis, and serum viscosity were performed. Human immunodeficiency virus (HIV) Ag/Ab testing was negative. Significant laboratory findings included beta-2 microglobulin = 10,980 μg/L, kappa light chain = 965 mg/dL, IgM = 7904 mg/dL, IgG = 414 mg/dL, and serum viscosity = 7.5 mPa⋅s; furthermore, the presence of marked lymphoplasmacytic cell infiltration on bone marrow biopsy confirmed the diagnosis of WM. The patient’s past medical history included hypertension controlled with antihypertensive medications, and a previous intracerebral hemorrhage resulting in residual right hemiparesis. The patient was a former smoker who had quit over 20 years ago. There was no documented history of diabetes, chronic kidney disease, or hereditary diseases.

The patient underwent multiple lines of chemotherapy from 2017 to 2019, including chlorambucil, bortezomib, and rituximab/cyclophosphamide/vincristine/methylprednisolone regimens, followed by maintenance rituximab (summarized in Fig. 1). He achieved a post-treatment serum IgM nadir of 801 mg/dL and maintained stable disease until late 2021.

**Figure 1.**
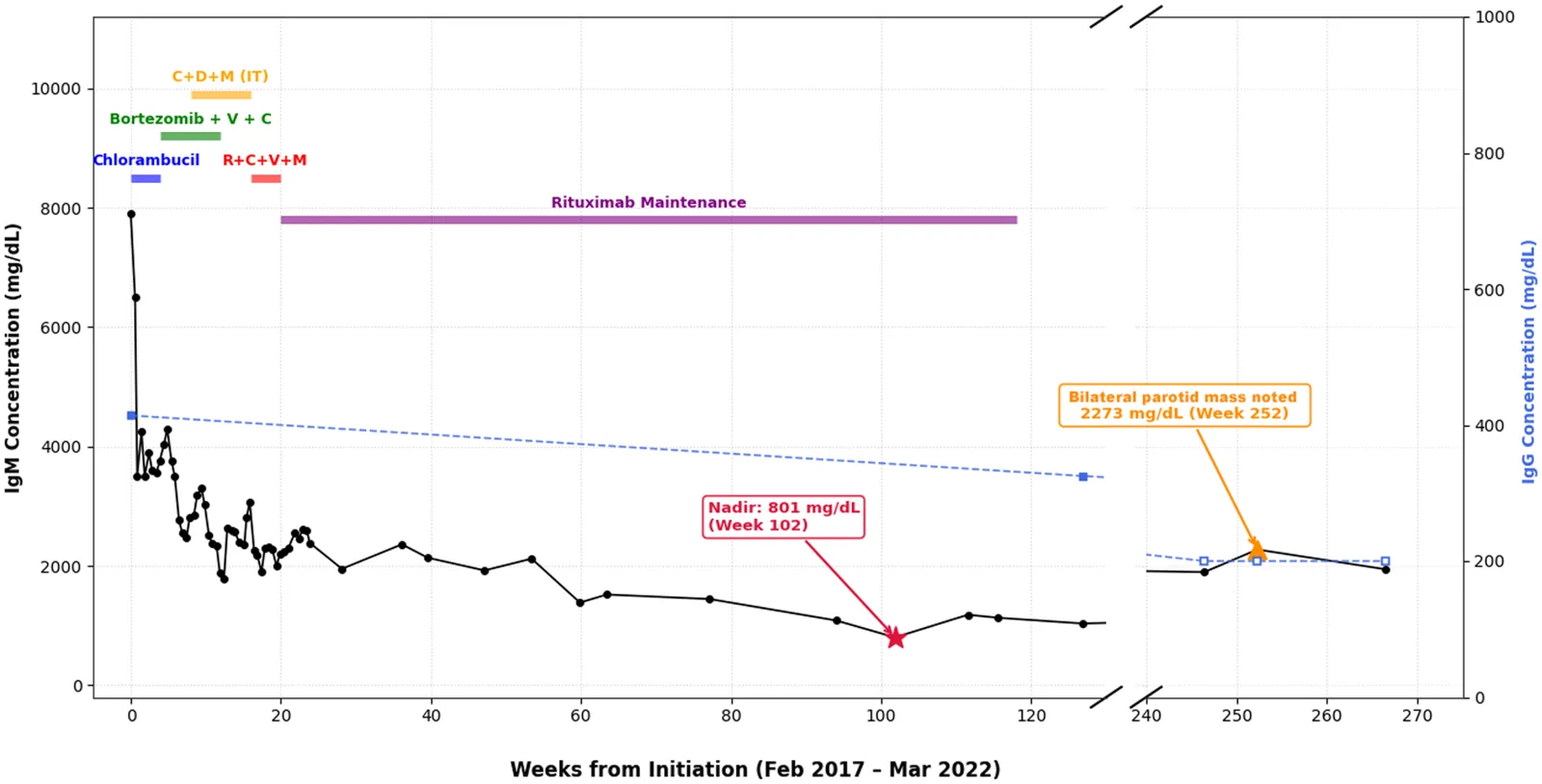
Longitudinal course of serum IgM and IgG levels and sequential treatments. Solid circles represent serum IgM levels. Solid squares represent serum IgG levels, while open squares represent IgG levels < 200 mg/dL. C + D + M (IT) = cytarabine + dexamethasone + intrathecal methotrexate, IgG = immunoglobulin G, IgM = immunoglobulin M, R + C + V + M = rituximab + cyclophosphamide + vincristine + methylprednisolone, V + C = vincristine + cyclophosphamide.

In late 2021, at the age of 79 years, the patient presented with painful, enlarged bilateral parotid masses (Fig. 2). Within one month of this presentation, computed tomography and magnetic resonance imaging demonstrated swelling of both parotid glands accompanied by multiple cystic lesions (Fig. 3). A subsequent ultrasonography performed one week later showed a focal honeycomb appearance in the bilateral parotid glands, and fine-needle aspiration (FNA) was performed. The cytology indicated atypia of undetermined significance but showed a monotonous proliferation of small- to medium-sized lymphocytes with lymphohistiocytic aggregates, while tingible body macrophages were scarce. Polymerase chain reaction (PCR) analysis was positive for immunoglobulin heavy chain-A (IgH-A), IgH-B, IgH-C, and IgH-D gene clonal rearrangements, but negative for IgH-E. Given the patient’s poor clinical status, with an Eastern Cooperative Oncology Group performance status of 4 at the time when extramedullary relapse was suspected, neither lymph node excisional biopsy nor bone marrow biopsy was performed. Laboratory evaluations revealed IgM = 2273 mg/dL, IgG < 200 mg/dL, hypocalcemia (serum calcium = 7.83 mg/dL), preserved renal function, and a serum viscosity level within the normal range. In addition, computed tomography and magnetic resonance imaging showed no evidence of osteolytic bone lesions. Based on the patient’s medical history and elevated serum IgM levels, the findings were most consistent with relapsed WM with EMD. The patient expired due to sepsis 4 months after the presentation of parotid enlargement.

**Figure 2.**
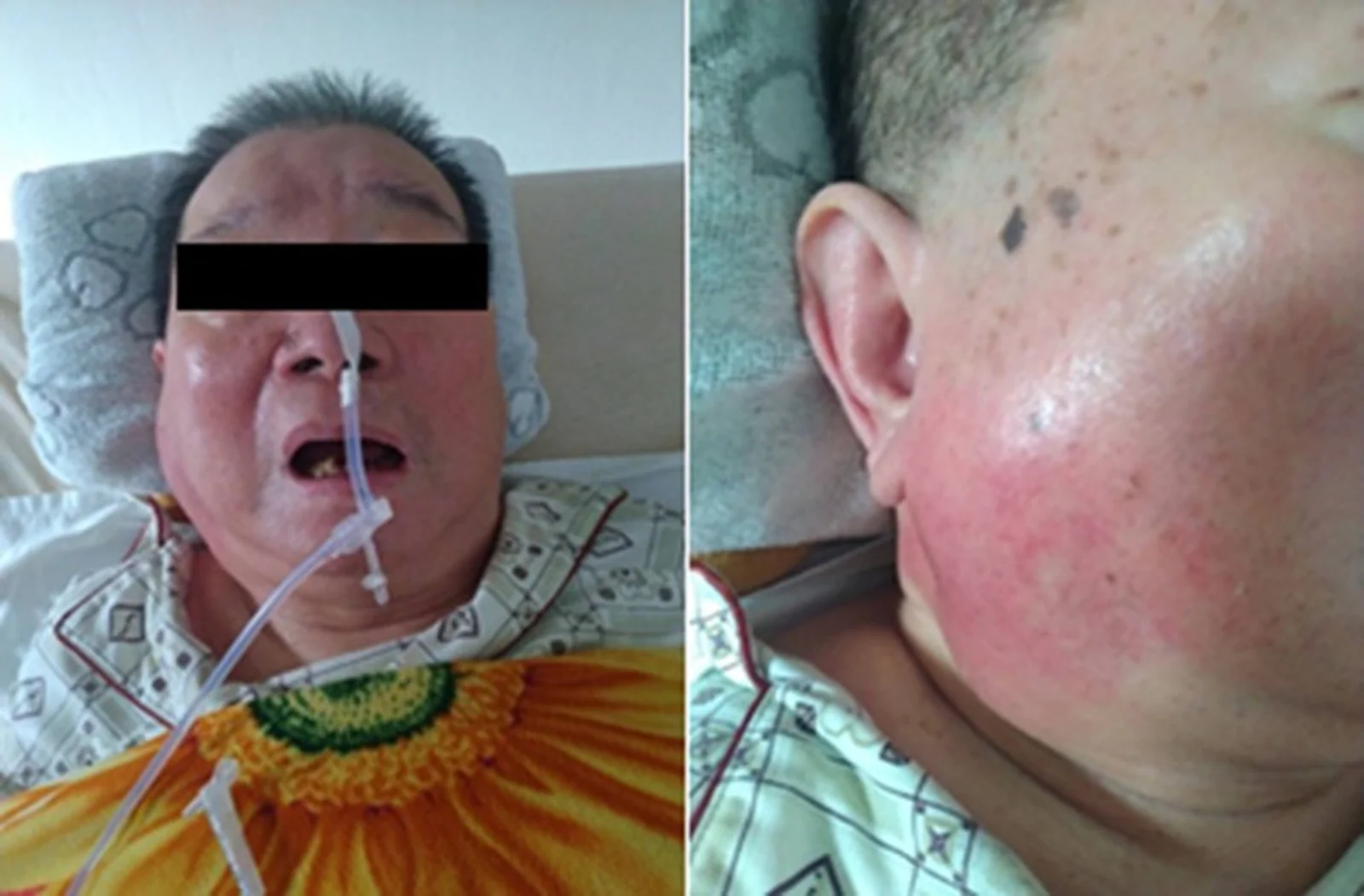
Clinical appearance of bilaterally enlarged parotid glands. The photographs were taken 1 mo after the diagnosis of parotid involvement and approximately 3 mo before death.

**Figure 3.**
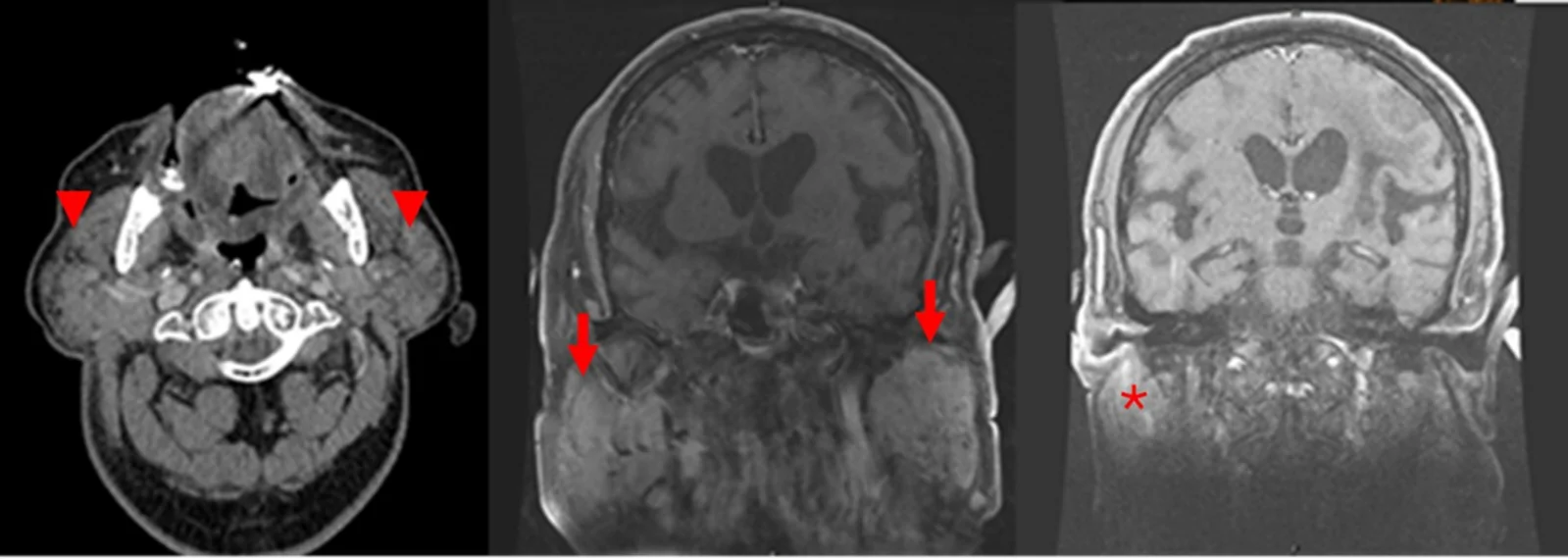
Radiographic comparison of parotid involvement. (Left) Axial CT with contrast shows bilateral parotid gland enlargement (arrowheads). (Middle) Coronal contrast-enhanced T1-weighted MRI confirms the enlargement, suspicious for WM relapse (arrows). (Right) Normal appearance of parotid gland during chemotherapy (asterisk). CT = computed tomography, MRI = magnetic resonance imaging, WM = Waldenström macroglobulinemia.

## 3. Discussion

WM is an indolent non-Hodgkin lymphoma characterized by lymphoplasmacytic infiltration of the bone marrow and the presence of circulating monoclonal IgM. In this report, we describe an elderly patient who experienced prolonged disease stability following multiple lines of chemotherapy and maintenance rituximab. Several years later, the patient developed rapidly progressive bilateral parotid gland enlargement, which was highly suggestive of relapsed WM with EMD. Extramedullary disease in WM is uncommon, with previous studies reporting an incidence of approximately 4.4 to 6.4%. The most frequently involved sites include the lungs, soft tissue, cerebrospinal fluid, kidneys, and bones. In most cases, EMD develops later in the disease course rather than being present at the initial diagnosis.^[2,3]^ To date, suspected relapsed WM with EMD presenting as bilateral cystic lesions of the parotid glands appears to be extremely rare. Although lymphoplasmacytic lymphoma (LPL) involving the parotid gland has been reported (Table 1),^[4,5]^ to our knowledge, this appears to be among the first reported cases of suspected relapsed WM with EMD presenting as bilateral cystic parotid involvement and accompanied by markedly elevated IgM levels.

**Table 1:** Clinical and pathological characteristics of reported cases of parotid gland lymphoplasmacytic lymphoma and the current case.

	Age/Sex	Cytologic diagnosis	Histologic diagnosis	Treatment	Survival (months)	Comments
Present case	79/M	LPD	N/A	Chemotherapy	62	History of WM
Chhieng et al,^[4]^ 2000	60/M	ML	LPL	N/A	N/A	History of nodal ML
von Stritzky et al,^[5]^ 1998	80/F	N/A	LPL	N/A	96	N/A
von Stritzky et al,^[5]^ 1998	92/M	Large cells	LPL	Chemotherapy	4	N/A

For the present case, age refers to the age at parotid presentation, treatment refers to therapy administered before the diagnosis of parotid involvement, and survival was calculated from the time of initial WM diagnosis. Data from previously published cases are presented as reported in the original publications.

LPD = lymphoproliferative disorder, LPL = lymphoplasmacytic lymphoma, ML = malignant lymphoma, mo = months, N/A = not available, WM = Waldenström macroglobulinemia.

The diagnosis of relapsed WM with EMD can be challenging and requires the integration of laboratory findings with clinicopathologic features. In the present case, relapse was suspected based on a marked increase in serum IgM levels of more than 25% from the nadir (exceeding 500 mg/dL),^[6]^ in accordance with established response criteria. The treatment-free interval of approximately 30 months further reflected the indolent nature of the disease. Despite this, the patient’s subsequent deterioration appeared to be driven by multiple overlapping clinical vulnerabilities rather than EMD alone, including advanced age, poor functional status, frailty, profound hypogammaglobulinemia with immunosuppression, and several comorbid conditions complicated by sepsis. Importantly, the prognostic impact of EMD in WM remains controversial in the current literature; historical cohorts suggest an aggressive course,^[7]^ whereas recent data show no significant survival detriment.^[2]^ Given these conflicting findings, causal attribution should remain cautious.

Although bilateral parotid gland enlargement may suggest inflammatory or infectious parotid disease, the absence of infectious signs, the lack of support for reactive lymphadenopathy on FNA, and the overall clinical, laboratory, imaging, and molecular findings made these diagnoses less likely. Other differential diagnoses include HIV-associated benign lymphoepithelial cysts, Sjögren syndrome/lymphoepithelial sialadenitis, IgG4-related disease, benign salivary gland lesions, metastatic disease, or other B-cell lymphomas. HIV infection was considered in the differential diagnosis; however, HIV Ag/Ab testing performed at the time of the initial WM diagnosis was negative. Sjögren syndrome and IgG4-related disease were considered retrospectively based on review of the available clinical records rather than being specifically evaluated at the time of the parotid presentation. No documented sicca symptoms or other clinical features suggestive of Sjögren syndrome were identified, and no clinical findings strongly supportive of IgG4-related disease were documented. Due to the monotonous proliferation of small- to medium-sized lymphocytes revealed by FNA, the differential diagnosis was directed toward a low-grade lymphoproliferative disorder (LPD) rather than benign salivary gland lesions or metastatic diseases; therefore, IgH PCR analysis was warranted to confirm clonal rearrangement.^[3]^

The IgH PCR we used in this case was the Biomedicine and Health Programme 2 assay. The Biomedicine and Health Programme 2 assay uses 5 complementary primer sets (IgH-A through IgH-E) targeting different regions of the IgH gene to maximize the detection of clonal B-cell populations despite biological heterogeneity. Positivity in 4 of 5 tubes (IgH-A, B, C, D) strongly supports the presence of a clonal B-cell population, consistent with the pathologist’s interpretation of LPD. The negative IgH-E result does not diminish diagnostic confidence when other tubes are positive.^[8]^ While IgH clonality testing alone does not independently establish a diagnosis of WM/LPL, it provides strong molecular evidence of B-cell clonality that must be interpreted within the overall clinicopathological context while recognizing residual diagnostic uncertainty. The differential diagnosis for LPD includes LPL, follicular lymphoma, mucosa-associated lymphoid tissue lymphoma, and IgM multiple myeloma. However, follicular lymphoma and mucosa-associated lymphoid tissue lymphoma are less likely due to significantly elevated IgM levels.^[9]^ Furthermore, the absence of osteolytic lesions, hypercalcemia, or renal impairment makes IgM multiple myeloma less probable. These findings are supportive rather than independently exclusionary. WM is classified as a type of LPL, especially when bone marrow involvement and circulating monoclonal IgM are observed. Importantly, a marked elevation of serum IgM from its post-treatment nadir, accompanied by severe secondary hypogammaglobulinemia (IgG < 200 mg/dL), reflects active clonal re-expansion and concomitant humoral immunosuppression. Based on the patient’s medical history, changes in IgM levels over time, imaging results, and molecular evidence of B-cell clonality, relapsed WM with EMD was considered the most likely diagnosis.

There is currently no standard treatment for relapsed WM, particularly in patients with poor performance status requiring palliative care. Later-line treatment options may include Bruton tyrosine kinase inhibitors, novel immunomodulatory agents, or autologous stem cell transplantation in selected patients.^[1]^ In the present case, following multiple lines of chemotherapy, maintenance rituximab achieved stable disease (defined as <25% increase and <25% reduction in IgM level)^[6]^ until relapse of WM. Previous studies showed that rituximab as a single agent has 40 to 50% overall response rates,^[10,11]^ suggesting that rituximab monotherapy is a treatment option, particularly for palliative purposes. For this patient, Bruton tyrosine kinase inhibitor therapy was avoided due to a prior intracranial hemorrhage, which represents a relative contraindication owing to the drug’s bleeding propensity, as well as the strict reimbursement criteria of the National Health Insurance. Further anti-WM therapy was withheld after relapse, aligning with the patient’s preference for palliative management, clinical frailty, and socioeconomic considerations regarding medical costs. Although the patient eventually succumbed to sepsis, his family expressed gratitude for the palliative approach that prioritized his comfort and quality of life during the final stages of the disease, avoiding aggressive and potentially toxic interventions.

A limitation of this study is the absence of histopathological confirmation due to the patient’s poor performance status. Although FNA cytology was performed, it showed atypia of undetermined significance rather than a definitive diagnosis of lymphoma, requiring cautious interpretation. Additionally, the lack of MYD88 L265P mutation testing, flow cytometric immunophenotyping after relapse, and repeat bone marrow biopsy, due to a combination of financial constraints and the patient’s transition to palliative care, leaves residual diagnostic uncertainty. Although single-case reports have inherent limitations regarding generalizability, the combination of clinical history, laboratory findings, imaging studies, and molecular evidence of B-cell clonality provided supportive evidence for relapsed WM as the most likely diagnosis.

## 4. Conclusion

In summary, suspected relapsed WM with EMD involving the parotid glands is extremely uncommon. In patients with a history of WM, newly developed bilateral cystic parotid enlargement should prompt consideration of extramedullary relapse while maintaining an appropriate differential diagnosis that includes benign lymphoepithelial cysts, Sjögren syndrome, IgG4-related disease, and other salivary gland disorders.

### Acknowledgments

We extend our special thanks to Hsin-Hua Lin for her kind assistance with reference management and software tools.

## Author contributions

**Conceptualization:** Hsin-Yao Hung, Tsung-Yu Yen.

**Investigation:** Yu-Cheng Chang, Tsung-Yu Yen.

**Supervision:** Tsung-Yu Yen.

**Writing – original draft:** Hsin-Yao Hung.

**Writing – review & editing:** Tsung-Yu Yen.
